# Machine learning-based MPO and Lp-PLA2 profiling reveals asthma-predominant inflammatory signature in asthma-COPD overlap^☆^

**DOI:** 10.1016/j.waojou.2026.101419

**Published:** 2026-07-02

**Authors:** Mingtao Liu, Jiaxi Chen, Jiani Yao, Yueying Zheng, Xiangyu Li, Haoming Zhong, Jiahui Liu, Jiating Zheng, Rui Ye, Huimin Huang, Peiyan Zheng, Baoqing Sun

**Affiliations:** a Department of Clinical Laboratory, State Key Laboratory of Respiratory Disease, National Center for Respiratory Medicine, National Clinical Research Center for Respiratory Disease, Guangzhou Institute of Respiratory Health, The First Affiliated Hospital of Guangzhou Medical University, Guangzhou 510120, China; b Guangzhou National Laboratory, Guangzhou 510005, China; c Guangdong Provincial Clinical Research Center for Laboratory Medicine, Guangzhou 510260, China; d Medical laboratory technology, KingMed School of Laboratory Medicine, Guangzhou Medical University, Guangzhou 511495, China; e Department of Clinical Medicine, The First Clinical College of Guangzhou Medical University, Guangzhou 511495, China; f Medical laboratory technology, The First Clinical Medical College of Guangdong Pharmaceutical University, Guangzhou 510006, China

**Keywords:** Asthma–COPD overlap syndrome, Machine learning, Myeloperoxidase, Lipoprotein–associated phospholipase A2, Inflammatory endotype

## Abstract

**Background:**

Asthma–COPD overlap (ACO) remains poorly characterized at the molecular level, leading to diagnostic uncertainty and suboptimal treatment. We hypothesized that machine learning analysis of myeloperoxidase (MPO) and lipoprotein–associated phospholipase A2 (Lp–PLA2) could help reveal ACO as a distinct inflammatory endotype.

**Methods:**

We prospectively enrolled 609 patients with obstructive airway diseases (asthma n = 255, COPD n = 100, AECOPD n = 182, ACO n = 72) at First Hospital of Guangzhou Medical University (National Center for Respiratory Medicine) between July 2023 and August 2025. Serum MPO and Lp–PLA2 were quantified using chemiluminescence immunoassay. Five machine learning algorithms (XGBoost, LightGBM, CatBoost, Neural Network, Random Forest) were employed for supervised classification, while unsupervised clustering (hierarchical, k–means, DBSCAN) defined molecular phenotypes. Model interpretability utilized SHAP analysis and dimensionality reduction techniques (PCA, UMAP, t–SNE).

**Results:**

MPO/Lp–PLA2 ratio emerged as a powerful discriminatory biomarker, with ACO patients exhibiting nominally lower ratios (1.66, IQR 1.19–2.27) resembling asthma (1.47, IQR 1.07–2.06) rather than COPD (2.26, IQR 1.98–2.56, FDR–adjusted *q* = 0.031). Unsupervised clustering revealed that 72.2% of clinically diagnosed ACO patients clustered with asthma, challenging traditional disease conceptualization. Ensemble machine learning model achieved exceptional performance (AUC 0.954, 95% CI: 0.926–0.982), significantly outperforming individual biomarkers (MPO/Lp–PLA2: AUC 0.782/0.714) and traditional clinical criteria (AUC 0.694). Clinically applicable ACORN (ACO recognition by neutrophil–eosinophil) score demonstrated 91.2% positive predictive value (PPV).

**Conclusion:**

Machine learning reveals ACO exhibits predominantly asthma–like inflammatory characteristics. MPO/Lp–PLA2 ratio and ACORN score enable precision phenotyping with potential therapeutic implications, advocating for biomarker–guided treatment strategies in obstructive airway diseases.

## Introduction

Asthma–COPD overlap (ACO) represents one of the most controversial entities in respiratory medicine, affecting 15–25% of patients with obstructive airway disease yet lacking clear molecular definition.^1^^,^^2^ While asthma and chronic obstructive pulmonary disease (COPD) have traditionally been distinguished by their inflammatory patterns, namely type 2 eosinophilic inflammation in asthma versus neutrophilic inflammation in COPD, ACO challenges this binary classification.^3^ Recent single–cell RNA sequencing studies have revealed distinct cellular populations in ACO patients, suggesting unique pathobiological mechanisms beyond simple disease coexistence.^4^ Despite the 2024 GOLD report acknowledging ACO's complexity, molecular criteria for diagnosis remain absent, resulting in inconsistent classification and suboptimal treatment outcomes.^5^^,^^6^ This diagnostic ambiguity has profound implications that ACO patients experience more frequent exacerbations, accelerated lung function decline, and higher healthcare utilization than those with asthma or COPD alone, yet evidence–based treatment guidelines remain elusive.^7^, ^8^, ^9^

Emergence of machine learning (ML) has revolutionized disease classification by enabling unbiased pattern recognition in complex biological data. In respiratory medicine, unsupervised clustering algorithms have identified 6 molecular asthma endotypes with distinct treatment responses, while deep learning models predict COPD progression with remarkable accuracy using routine clinical data.^10^^,^^11^ However, the application of ML to molecularly characterize ACO remains unexplored, representing a critical gap in precision pulmonology.

Two inflammatory biomarkers, myeloperoxidase (MPO) and lipoprotein–associated phospholipase A2 (Lp–PLA2), offer particular promise for molecular phenotyping. MPO, a heme peroxidase predominantly expressed in neutrophils, catalyzes hypochlorous acid formation with serum levels strongly correlating with sputum MPO in COPD patients.^12^^,^^13^ Recent proteomics revealed MPO interacts with multiple proteins in neutrophil extracellular trap networks, amplifying inflammatory cascades through self–sustaining oxidative cycles when concentrations exceed critical thresholds.^14^ Lp–PLA2 is a phospholipase carried predominantly on circulating lipoproteins and is best established as a marker of vascular inflammation.^15^ Evidence linking Lp–PLA2 to airway disease is emerging rather than definitive: elevated Lp–PLA2 activity has been reported in non–severe asthma,^16^ and higher circulating Lp–PLA2 has recently been associated with emphysema and airflow obstruction in COPD,^17^ supporting further investigation of this marker in obstructive airway phenotyping.

We hypothesized that ML analysis of MPO and Lp–PLA2 signatures could indicate ACO as a distinct inflammatory endotype rather than a simple overlap of asthma and COPD. This study employed both supervised and unsupervised ML algorithms to analyze biomarker patterns across 609 patients with obstructive airway disease. Our objectives were to determine whether ACO exhibits a unique MPO–Lp–PLA2 signature distinguishable from asthma, COPD, and acute exacerbations; develop an interpretable ML model for accurate ACO classification; and validate whether ML–defined ACO demonstrates distinct clinical characteristics. By integrating molecular biomarkers with artificial intelligence, we aimed to transform ACO from a problematic overlap syndrome into a precisely defined therapeutic target.

## Methods

### Study design and patient selection

This prospective cross–sectional study was conducted at First Hospital of Guangzhou Medical University (National Center for Respiratory Medicine) between July 2023 and August 2025. Study protocol received approval from the Institutional Review Board (approval number: GYFYY–2022–135) and complied with the Declaration of Helsinki principles. All participants provided written informed consent prior to enrollment.

From an initial cohort of 1082 patients with obstructive airway diseases, 471 were excluded due to incomplete clinical records (n = 361) or missing diagnostic criteria (n = 110), yielding 609 eligible participants. Patients were stratified into 4 groups based on established international guidelines: (1) Asthma (n = 255, 42%), diagnosed per Global Initiative for Asthma (GINA) 2023 criteria, requiring variable respiratory symptoms and reversible airflow limitation (post–bronchodilator forced expiratory volume in 1 s (FEV_1_) increase ≥12% and ≥200 mL); (2) COPD (n = 100, 16%), diagnosed per Global Initiative for Chronic Obstructive Lung Disease (GOLD) 2023 guidelines, characterized by persistent symptoms, relevant exposure history, and post–bronchodilator FEV_1_/forced vital capacity (FVC). ACO was defined according to the 2023 consensus criteria:^15^ patients ≥40 years with persistent airflow limitation (post–bronchodilator FEV_1_/FVC <0.7), significant bronchodilator reversibility (≥12% and ≥400 mL FEV_1_ increase), and either blood eosinophils ≥300 cells/μL or FeNO ≥50 ppb, with documented history of both asthma and COPD features. Sample size was determined using G∗Power 3.1.9.7, with 72 ACO patients providing 85% power to detect a 0.5 standard deviation difference in biomarker levels between groups (α = 0.05, two–tailed), aligning with prior ACO research.^16^

### Clinical assessments and laboratory procedures

Baseline evaluations comprised a detailed medical history, including smoking status and exposure history, and a physical examination focusing on respiratory signs. Standardized questionnaires assessed disease–specific outcomes: Asthma Control Test (ACT) for asthma, COPD Assessment Test (CAT) for COPD, and modified Medical Research Council (mMRC) dyspnea scale for symptom severity. Pulmonary function testing (PFT) was performed using the MasterScreen™ PFT System (Jaeger, Germany), adhering to American Thoracic Society/European Respiratory Society (ATS/ERS) standards,^17^ to measure FEV_1_, FVC, and reversibility. Venous blood samples (5 mL) were collected after overnight fasting into serum separator tubes. Samples underwent centrifugation within 2 h at 3000 rpm for 10 min at room temperature, followed by aliquoting into 1 mL cryovials and storage at −80 °C to preserve biomarker integrity. Complete blood counts, including total and differential leukocyte counts, neutrophil–to–lymphocyte ratio (NLR), and eosinophil percentage, were analyzed using the Beckman Coulter AU5821 automated hematology analyzer (Beckman Coulter, Inc., Brea, CA, USA) analyzer.

Sputum induction was performed according to standardized ERS–based procedures.^18^ After premedication with inhaled salbutamol (400 μg), subjects inhaled isotonic or hypertonic saline generated by an ultrasonic nebulizer in sequential inhalation periods under direct medical supervision. Spirometry or peak expiratory flow was monitored during the procedure when clinically indicated, and the induction was discontinued if the patient developed intolerance or if FEV_1_ fell by >20% from the post–bronchodilator baseline. Visible sputum plugs were selected and processed within 2 h using dithiothreitol, followed by filtration, centrifugation, and cytospin preparation for differential cell counting. Whenever sample quality permitted, at least 400 non–squamous cells were counted.

In patients with AECOPD, sputum induction was undertaken only when the treating physicians considered the patient clinically stable enough to tolerate the procedure. A modified protocol with bronchodilator pretreatment, close monitoring, and early termination criteria was used. When spontaneous sputum of acceptable cytologic quality was available, the same downstream processing protocol was applied without further induction.^19^

### Quantification of serum MPO and Lp–PLA2

Serum concentrations of MPO and Lp–PLA2 were determined using a high–sensitivity magnetic particle based chemiluminescence immunoassay (CLIA) with commercially available kits (Auto Bio Diagnostics Co., Ltd., Zhengzhou, China). This assay was performed on an Auto A2000plus automated immunoassay analyzer according to the manufacturer's instructions. Briefly, paramagnetic particles coated with monoclonal anti–MPO or anti–Lp–PLA2 antibodies were incubated with 200 μL of patient serum, followed by addition of acridinium ester–labeled secondary antibodies to form immune complexes. After magnetic separation and washing, the chemiluminescent signal was measured and quantified using a standard curve. The assay detection limits were 0.5 ng/mL for MPO and 1.0 ng/mL for Lp–PLA2, with intra–assay coefficients of variation (CVs) < 4.5% and inter–assay CVs <8.0% for both analytes. All measurements were performed in duplicate by laboratory personnel blinded to clinical information, with mean values used for statistical analysis. Quality control samples covering low, medium, and high concentration ranges were included in each assay run to ensure analytical precision and accuracy. The reference ranges established in our laboratory based on healthy controls were 20–65 ng/mL for MPO and 140–420 ng/mL for Lp–PLA2, consistent with previously published values.^20^^,^^21^

### Traditional biomarker measurements

Fractional exhaled nitric oxide (FeNO) was measured using the NIOX VERO® (Circassia, UK) according to ATS/ERS guidelines, with values ≥ 50 ppb indicating significant eosinophilic inflammation. Total IgE was measured using ImmunoCAP™ (Thermo Fisher Scientific) > 105 IU/mL means allergic positive reaction.

### Machine learning framework

#### Data preparation

Missing data (<5% across all variables) were imputed using Multiple Imputation by Chained Equations (MICE) with predictive mean matching for continuous variables and logistic regression for categorical variables.^22^ Outliers were detected using the Isolation Forest algorithm with contamination parameter set at 0.05, followed by manual clinical review to distinguish true outliers from extreme phenotypes. Feature scaling employed RobustScaler for biomarker variables to minimize the influence of outliers, and StandardScaler for normally distributed clinical variables. Multicollinearity was assessed using variance inflation factor (VIF), with features exhibiting VIF >5 excluded from subsequent analyses.

#### Feature engineering

Following established methodologies,^23^ we created biologically informed features including: (1) Ratio features: MPO/Lp–PLA2, MPO/eosinophil count, Lp–PLA2/neutrophil count, neutrophil–to–lymphocyte ratio; (2) Polynomial features: MPO^2^, Lp–PLA2^2^, and their interaction term (MPO × Lp–PLA2); (3) Domain–specific composite indices: Oxidative Stress Index = (MPO × neutrophil count)/(antioxidant capacity × lymphocyte count), Inflammatory Burden Score = weighted sum of standardized inflammatory markers; (4) Temporal features: rate of FEV_1_ decline (when available from medical records), annual exacerbation frequency.

#### Model development

We implemented an ensemble ML approach integrated 4 optimized base learners— XGBoost 2.0.3 (n_estimators = 1500, max_depth = 8, learning_rate = 0.01, reg_lambda = 1.2), LightGBM 4.1.0 (num_leaves = 31, min_child_samples = 20, feature_fraction = 0.8), CatBoost 1.2 (iterations = 2000, depth = 6, l2_leaf_reg = 3), and a neural network implemented in PyTorch 2.1 with architecture (128–64–32) neurons, ReLU activation, dropout = 0.3, and batch normalization. The meta–learner consisted of L2–regularized logistic regression combining base model predictions. Model hyperparameters were optimized using Bayesian optimization with 5–fold stratified cross–validation.

#### Interpretability analysis

Model interpretability was enhanced through SHAP (SHapley Additive exPlanations) analysis to rank feature importance,^24^ Uniform Manifold Approximation and Projection (UMAP) and t–distributed Stochastic Neighbor Embedding (t–SNE) visualizations to explore phenotypic clustering, and Local Interpretable Model–agnostic Explanations (LIME) to elucidate individual predictions.

#### ACORN score development

Leveraging ML insights, we developed the ACO Recognition by Neutrophil–eosinophil (ACORN) clinical score, a composite metric validated in a temporally separated cohort (n = 156) enrolled at the same center during a non–overlapping period using identical protocols, to stratify ACO risk.

### Statistical analysis

Traditional statistical analyses were performed using GraphPad Prism 9.3.0 and R 4.2.0. Data distribution was assessed using Shapiro–Wilk test and Q–Q plots. Non–parametric tests (Kruskal–Wallis with Dunn's post–hoc) were used for group comparisons given the non–normal distribution of most variables. Benjamini–Hochberg false discovery rate (FDR) procedure was applied for pairwise post–hoc comparisons of the MPO/Lp–PLA2 ratio, with *q* < 0.05 considered significant. Machine learning model stability was evaluated through bootstrap validation (n = 1000 iterations) with performance metrics reported as mean ± 95% confidence intervals. Feature importance rankings were validated using permutation testing (n = 10,000 iterations) with Bonferroni correction for multiple comparisons. Clinical utility of the ML–based classification was assessed using decision curve analysis, comparing net benefit across probability thresholds.^25^ Model calibration was evaluated using calibration plots and the Hosmer–Lemeshow test. For all analyses, two–sided *p*–values <0.05 were considered statistically significant.

## Results

### Study population and baseline characteristics

**Fig. 1:**
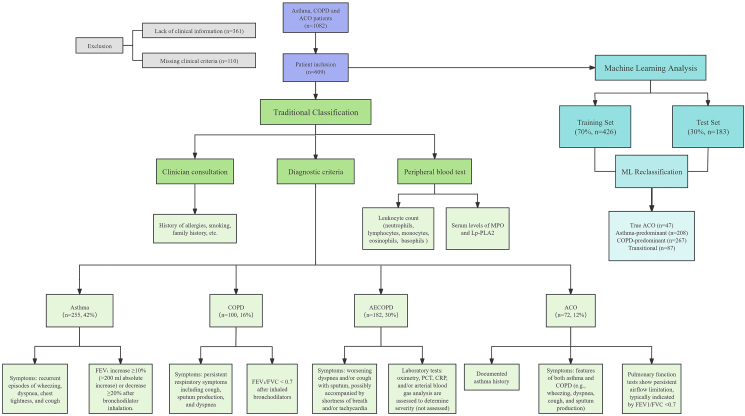
**Study design and patient enrollment flowchart**. This flowchart outlines the process of patient inclusion, traditional classification, and machine learning (ML) reclassification for asthma, chronic obstructive pulmonary disease (COPD), and asthma–COPD overlap (ACO) patients. After excluding 471 patients due to missing clinical information or criteria, 609 patients were included. Traditional classification involved clinician consultations, diagnostic criteria based on symptoms (eg, wheezing, cough, and FEV_1_/FVC values), and peripheral blood tests (leukocyte count, serum levels of MPO and Lp–PLA2). Patients were categorized into asthma (n = 255, 42%), COPD (n = 100, 16%), AECOPD (n = 182, 30%), and ACO (n = 72, 12%) based on these criteria. Machine learning analysis, splitting the cohort into training (70%) and test (30%) sets, reclassified patients into 4 groups: true ACO, asthma–predominant, COPD–predominant, and transitional, which highlights the integration of traditional diagnostic methods with ML techniques to refine classification and improve diagnostic accuracy. **Abbreviations:** COPD: chronic obstructive pulmonary disease; AECOPD: acute exacerbation of chronic obstructive pulmonary disease; ACO: asthma–COPD overlap; MPO: myeloperoxidase; Lp–PLA2: lipoprotein–associated phospholipase A2; FEV_1_: forced expiratory volume in 1 s; FVC: forced vital capacity; ML: machine learning

We enrolled 609 patients with obstructive airway diseases between July 2023 and August 2025 at First Hospital of Guangzhou Medical University (National Center for Respiratory Medicine). This cohort comprised 4 distinct phenotypes: asthma (n = 255, 41.8%), COPD (n = 100, 16.4%), acute exacerbation of COPD (AECOPD; n = 182, 29.9%), and asthma–COPD overlap (ACO; n = 72, 11.8%). This represents 56.3% of the initial screening population (n = 1082), with 471 patients excluded due to incomplete clinical records (n = 361) or failure to meet diagnostic criteria (n = 110) (Table 1, Fig. 1).

**Table 1:** Demographic and clinical characteristics of study population.

Characteristics	Asthma (n = 255)	COPD (n = 100)	AECOPD (n = 182)	ACO (n = 72)	*P*–value
**Demographics**					
Age, years	49 (35–80)	66 (59–74)	69 (62–75)	58 (52–66)	<0.001
Sex, male, n (%)	105 (41)	85 (85)	160 (88)	40 (56)	<0.001
BMI, kg/m^2^	24.2 (22.1–26.8)	22.8 (20.5–25.2)	21.9 (19.8–24.3)	23.5 (21.2–25.9)	0.012
**Smoking history**					
Never smoker, n (%)	232 (91)	65 (65)	43 (24)	46 (64)	<0.001
Ex–smoker, n (%)	9 (3)	25 (25)	73 (40)	17 (23)	<0.001
Current smoker, n (%)	14 (6)	10 (10)	66 (36)	9 (13)	<0.001
Pack–years	0 (0–0)	25 (15–40)	35 (20–50)	10 (0–25)	<0.001
**Clinical scores**					
ACT score	18 (15–21)	–	–	16 (13–19)	0.142
CAT score	–	18 (14–23)	24 (20–28)	20 (16–24)	0.003
mMRC scale	1 (0–2)	2 (1–3)	3 (2–4)	2 (1–3)	<0.001
Exacerbations/year	0.5 (0–1)	1.5 (1–2)	3.2 (2–4)	2.1 (1–3)	<0.001
**Novel biomarkers**					
MPO, ng/mL	136.8 (71.9–266.3)	165.5 (118.8–250.7)	327.9 (212.3–457.2)	164.2 (55.9–264.5)	<0.001
Lp–PLA2, ng/mL	93.1 (67.2–129)	131.6 (107.3–171.1)	134.9 (104.5–173.9)	98.9 (74.3–131.5)	0.002
MPO/Lp–PLA2 ratio	1.47 (1.07–2.06)	2.26 (1.98–2.56)	2.43 (1.96–3.05)	1.66 (1.19–2.27)	0.008
**Traditional biomarkers**					
FeNO, ppb	45 (28–72)	18 (12–25)	16 (10–23)	38 (25–55)	<0.001
IgE total, IU/mL	285 (125–520)	68 (35–115)	72 (38–128)	195 (98–385)	0.006
**Blood cell counts**					
Neutrophil, 10^9^/L	4.03 (3.03–5.5)	4.3 (3.45–5.72)	4.67 (3.67–7.08)	4.67 (3.64–6.73)	0.021
Lymphocyte, 10^9^/L	2.00 (1.63–2.42)	1.83 (1.45–2.19)	1.30 (0.96–1.8)	1.71 (1.16–2.21)	0.003
Eosinophil, 10^9^/L	0.31 (0.15–0.62)	0.18 (0.11–0.31)	0.15 (0.05–0.25)	0.23 (0.1–0.41)	0.009
Eosinophil, %	4.8 (2.3–8.2)	2.1 (1.2–3.5)	1.8 (0.8–2.9)	3.5 (2.0–5.8)	0.012
NLR	2.02 (1.51–2.89)	2.35 (1.82–3.15)	3.59 (2.65–5.21)	2.73 (2.14–3.82)	0.007
**Pulmonary function testing**					
FVC, % predicted	93.3 (81.1–103)	81.6 (67.8–95.8)	72.2 (58.0–87.1)	80.6 (69.6–92.6)	<0.001
FEV_1_, % predicted	79.3 (58.4–90.6)	42.0 (31.8–63.9)	37.9 (27.7–60.5)	51.9 (37.8–64.3)	<0.001
FEV_1_/FVC, %	77.5 (72.3–82.1)	52.3 (45.2–61.5)	50.8 (43.1–59.7)	58.4 (51.2–65.8)	<0.001
**Sputum Cytology**					
Neutrophil, %	58.1 (49.4–64)	62.3 (54.8–69.9)	69.0 (61.2–78.4)	62.0 (55.0–78.0)	0.018
Eosinophil, %	4.5 (1.9–9.0)	2.4 (1.6–4.3)	2.0 (0.5–3.9)	3.1 (1.2–5.3)	0.024

Data presented as median (IQR) or n (%). P–values from Kruskal–Wallis test for continuous variables and Chi–square test for categorical variables.

Abbreviations: COPD: chronic obstructive pulmonary disease; AECOPD: acute exacerbation of chronic obstructive pulmonary disease; ACO: asthma–COPD overlap; BMI: body mass index; ACT: Asthma Control Test; CAT: COPD Assessment Test; mMRC: modified medical research council; MPO: myeloperoxidase; Lp–PLA2: lipoprotein–associated phospholipase A2; FeNO: fractional exhaled nitric oxide; IgE: immunoglobulin E; NLR: neutrophil–to–lymphocyte ratio; FEV1: forced expiratory volume in 1 s; FVC: forced vital capacity; BDR: bronchodilator response

Significant demographic heterogeneity characterized the 4 phenotypes. Patients with COPD (median age 66 years, IQR 59–74) and AECOPD (69 years, IQR 62–75) were significantly older than those with asthma (49 years, IQR 35–80) or ACO (58 years, IQR 52–66; *p* < 0.001 for all comparisons). Male predominance in COPD (85%) and AECOPD (88%) contrasted sharply with the more balanced gender distribution in asthma (41% male) and ACO (56% male), consistent with established epidemiological patterns.

Smoking exposure patterns demonstrated disease–specific stratification. Never–smokers comprised 91% of asthma patients, whereas AECOPD exhibited the highest tobacco burden with 76% ever–smokers (36% current, 40% former; *p* < 0.001). Intermediate smoking profile in ACO (64% never–smokers) supports its classification as a distinct clinical entity.

PFT assessments revealed characteristic obstructive physiology with disease–specific severity gradients. FEV_1_ percent predicted demonstrated progressive deterioration: asthma (79.3%, IQR 58.4–90.6), ACO (51.9%, IQR 37.8–64.3), COPD (42.0%, IQR 31.8–63.9), and AECOPD (37.9%, IQR 27.7–60.5; *p* < 0.001). Despite this severity gradient, FEV_1_/FVC ratios remained uniformly reduced across COPD, AECOPD, and ACO groups, confirming fixed airflow limitation as their unifying pathophysiological feature.

### Inflammatory biomarker signatures refine respiratory phenotypes

**Fig. 2:**
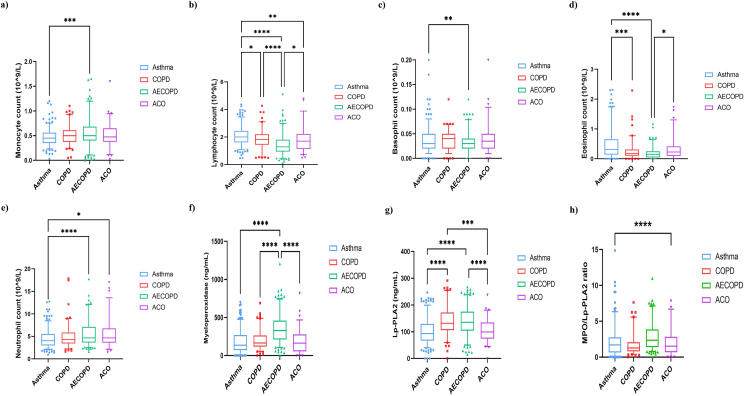
**Comparison of hematological parameters across asthma, COPD, AECOPD, and ACO groups**. This figure shows box plots comparing various peripheral blood test parameters across 4 groups: asthma (blue), COPD (red), AECOPD (green), and ACO (purple). **a)** Monocyte counts showed mild elevation in AECOPD compared to asthma (p < 0.001). **b)** Lymphocyte counts were significantly reduced in AECOPD compared to all other groups (p < 0.0001 vs asthma and COPD; p < 0.05 vs ACO), consistent with stress–induced lymphopenia during acute exacerbations. **c)** Basophil counts were comparable across groups with only AECOPD showing mild reduction compared to asthma (p < 0.01). **d)** Eosinophil counts demonstrated a clear type 2 inflammatory gradient: asthma > ACO > COPD > AECOPD, with significant differences between asthma and both COPD phenotypes (p < 0.001 and p < 0.0001). **e)** Neutrophil counts were elevated in AECOPD compared to asthma (p < 0.0001) and ACO (p < 0.05), reflecting neutrophil–predominant inflammation. **f)** Serum MPO concentrations showed marked elevation in AECOPD (median 327.9 ng/mL) compared to all other phenotypes (p < 0.0001). **g)** Lp–PLA2 levels were significantly higher in COPD phenotypes compared to asthma (p < 0.0001) and ACO (p < 0.001 for AECOPD vs ACO). **h)** MPO/Lp–PLA2 ratio showed significant overall variation (p < 0.0001 by Kruskal–Wallis test), with AECOPD displaying the highest ratio, though post–hoc pairwise comparisons did not reach statistical significance after correction for multiple testing. Data presented as box plots showing median (line), interquartile range (box), 1.5 × IQR (whiskers), and individual outliers (dots). Statistical analysis performed using Kruskal–Wallis test with Dunn's post–hoc correction. ∗p < 0.05, ∗∗p < 0.01, ∗∗∗p < 0.001, ∗∗∗∗p < 0.0001. **Abbreviations:** AECOPD: acute exacerbation of chronic obstructive pulmonary disease; COPD: chronic obstructive pulmonary disease; ACO: asthma–COPD overlap; MPO: myeloperoxidase; Lp–PLA2: lipoprotein–associated phospholipase A2

Comprehensive inflammatory profiling revealed distinct immunological signatures that differentiated respiratory phenotypes with high precision (Fig. 2). Peripheral blood neutrophil counts peaked in AECOPD (4.67 × 10^9^/L, IQR 3.67–7.08) compared to asthma (4.03 × 10^9^/L, IQR 3.03–5.5; *p* < 0.0001) (Fig. 2e), while lymphocyte counts showed inverse patterns with marked suppression in AECOPD (1.30 × 10^9^/L, IQR 0.96–1.80) versus asthma (2.00 × 10^9^/L, IQR 1.63–2.42; *p* < 0.0001) (Fig. 2b), consistent with stress–induced lymphopenia during acute exacerbations. Eosinophil counts were significantly elevated in asthma (0.31 × 10^9^/L, IQR 0.15–0.62) compared to AECOPD (0.15 × 10^9^/L, IQR 0.05–0.25; *p* < 0.0001), and also compared to COPD (0.18 × 10^9^/L, IQR 0.11–0.31; *p* < 0.001), confirming predominant type 2 inflammation in asthma pathogenesis (Fig. 2d).

Serum MPO levels revealed a striking inflammatory gradient across phenotypes, with AECOPD exhibiting significantly higher concentrations (327.9 ng/mL, IQR 212.3–457.2) compared to COPD (165.5 ng/mL, IQR 118.8–250.7; *p* < 0.0001), asthma (136.8 ng/mL, IQR 71.9–266.3; *p* < 0.0001), and ACO (164.2 ng/mL, IQR 55.9–264.5; *p* < 0.0001) (Fig. 2f). This pronounced elevation in AECOPD likely reflects progressive neutrophil activation and degranulation, with MPO–mediated hypochlorous acid production driving oxidative tissue injury during exacerbations.

Critically, the MPO/Lp–PLA2 ratio emerged as a novel discriminatory biomarker. ACO patients exhibited nominally lower ratios (1.66, IQR 1.19–2.27) compared to COPD (2.26, IQR 1.98–2.56, FDR–adjusted *q* = 0.031), yet similar to asthma (1.47, IQR 1.07–2.06, *p* = 0.14) (Fig. 2h), suggesting ACO shares greater inflammatory similarity with asthma than COPD. This ratio ranked first in feature importance in our machine learning model (SHAP value: 0.284), underscoring its diagnostic utility (Table 3). While FeNO levels demonstrated a clear T2 inflammatory gradient: asthma (45 ppb, IQR 28–72) > ACO (38 ppb, IQR 25–55) > COPD (18 ppb, IQR 12–25) > AECOPD (16 ppb, IQR 10–23; *p* < 0.001), confirming the T2–high component in ACO patients.

**Table 2:** Machine learning model performance metrics for ACO classification.

Model	Accuracy (%)	Sensitivity (%)	Specificity (%)	AUC	PPV (%)	NPV (%)	F1–Score
**Individual models**							
XGBoost	94.2 (91.3–96.5)	87.3 (81.2–92.1)	91.1 (87.8–93.7)	0.943 (0.912–0.974)	89.2 (83.5–93.4)	89.8 (86.3–92.6)	0.882
Random Forest	92.8 (89.6–95.2)	85.1 (78.6–90.2)	89.7 (86.2–92.5)	0.927 (0.893–0.961)	87.3 (81.2–91.8)	88.1 (84.5–91.1)	0.861
LightGBM	93.5 (90.5–95.8)	86.2 (79.9–91.1)	90.3 (87.0–93.0)	0.936 (0.904–0.968)	88.1 (82.3–92.4)	88.9 (85.4–91.8)	0.871
Neural network	91.4 (88.0–94.1)	83.9 (77.2–89.2)	88.2 (84.5–91.2)	0.918 (0.882–0.954)	85.7 (79.3–90.6)	86.8 (83.1–89.9)	0.848
CatBoost	93.1 (90.0–95.5)	85.7 (79.4–90.6)	89.9 (86.5–92.7)	0.932 (0.899–0.965)	87.6 (81.6–92.0)	88.4 (84.9–91.3)	0.866
**Ensemble model**							
Stacked ensemble	95.1 (92.5–97.0)	88.9 (83.2–93.2)	92.4 (89.4–94.7)	0.954 (0.926–0.982)	90.6 (85.2–94.4)	91.2 (88.0–93.7)	0.897
**Traditional methods**							
Clinical criteria	71.3 (66.8–75.4)	62.5 (54.9–69.6)	68.9 (64.7–72.8)	0.694 (0.649–0.739)	64.2 (56.7–71.1)	67.3 (63.1–71.2)	0.633
MPO alone	78.2 (74.1–81.8)	70.1 (62.9–76.6)	75.3 (71.4–78.9)	0.782 (0.741–0.823)	71.8 (64.8–77.9)	73.7 (69.8–77.3)	0.709
Lp–PLA2 alone	71.4 (66.9–75.5)	65.3 (57.8–72.2)	69.1 (64.9–73.0)	0.714 (0.670–0.758)	65.8 (58.4–72.6)	68.6 (64.4–72.5)	0.655

Data presented as value (95% CI).

Abbreviations: ACO: asthma–COPD overlap; MPO: myeloperoxidase; Lp–PLA2: lipoprotein–associated phospholipase A2; AUC: area under the curve; PPV: positive predictive value; NPV: negative predictive value

Induced sputum differential cell counts provided complementary evidence of compartmentalized airway inflammation, with AECOPD demonstrating maximal neutrophil percentage (69%, IQR 61.2–78.4) compared to asthma (58.1%, IQR 49.4–64.0; *p <* 0.001) (Table 1), reinforcing the predominant neutrophilic inflammatory pattern in AECOPD. Conversely, eosinophil percentages were approximately two–fold higher in asthma (4.5%, IQR 1.9–9.0) versus COPD (2.4%, IQR 1.6–4.3; *p* < 0.001) and AECOPD (2.0%, IQR 0.5–3.9; *p* < 0.001), consistent with the established type 2 inflammatory signature in asthma. Furthermore, MPO/Lp–PLA2 ratio demonstrated a significant inverse correlation with FeNO levels across all phenotypes (Spearman r = −0.62, *p* < 0.001), confirming that this novel ratio captures the balance between neutrophilic and T2–high eosinophilic inflammation.

### Multidimensional analysis reveals ACO's inflammatory identity

**Fig. 3:**
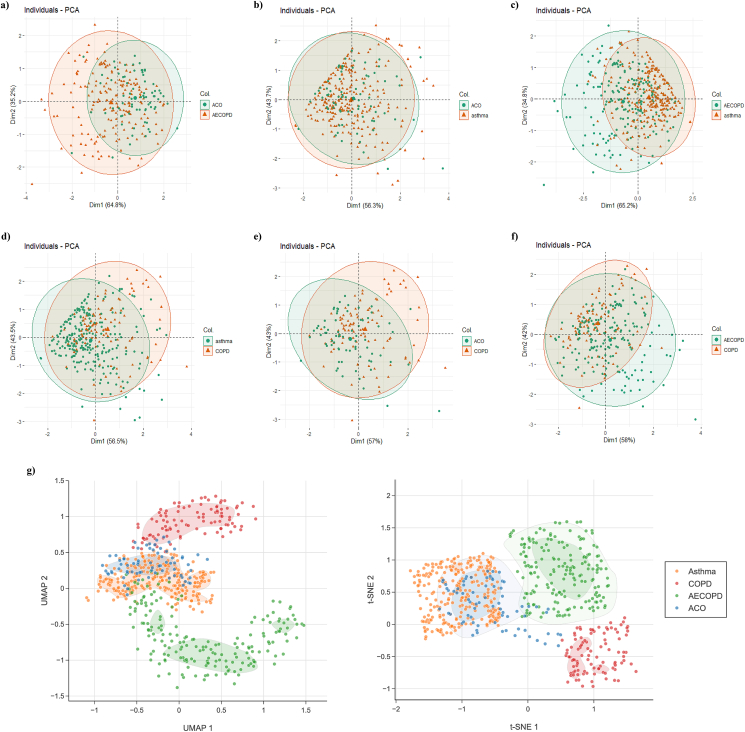
**Principal component analysis and dimensionality reduction visualizations of asthma, COPD, AECOPD, and ACO patients**. **a)** ACO vs AECOPD: despite both conditions involving airway obstruction, AECOPD shows more dispersed distribution along Dimension 1 (MPO axis). **b)** ACO vs asthma: substantial overlap between ACO and asthma clusters, with ACO patients predominantly clustering within the asthma distribution. **c)** Asthma vs AECOPD: clear separation between clusters. **d)** Asthma vs COPD: moderate separation with COPD showing intermediate inflammatory profile between asthma and AECOPD. **e)** ACO vs COPD: notable separation suggesting ACO inflammatory signature differs from stable COPD, aligning more closely with asthma patterns. **f)** AECOPD vs COPD: AECOPD demonstrates broader inflammatory distribution. Dimension 1 corresponds to MPO levels (explaining 56.3–65.2% of variance), while Dimension 2 represents Lp–PLA2 levels (explaining 34.8–43.7% of variance). **g)** UMAP (Uniform Manifold Approximation and Projection) and t–SNE (t–distributed stochastic neighbor embedding) visualizations confirm the PCA findings, demonstrating that ACO patients (blue) cluster predominantly with asthma (orange) rather than COPD phenotypes (red and green). n = 255 (asthma), 100 (COPD), 182 (AECOPD), 72 (ACO). **Abbreviations:** COPD: chronic obstructive pulmonary disease; AECOPD: acute exacerbation of chronic obstructive pulmonary disease; ACO: asthma–COPD overlap; MPO: myeloperoxidase; Lp–PLA2: lipoprotein–associated phospholipase A2; PCA: principal component analysis; UMAP: Uniform Manifold Approximation and Projection; t–SNE: t–distributed stochastic neighbor embedding

We performed principal component analysis (PCA) integrating MPO and Lp–PLA2 levels (Fig. 3a–f), which revealed distinct clustering patterns with significant pathophysiological implications. While AECOPD and ACO clusters showed partial overlap, AECOPD demonstrated greater dispersion along the MPO axis (Dimension 1, corresponding to MPO levels explaining 56.3–65.2% of variance; Dimension 2 represents Lp–PLA2 levels, explaining 34.8–43.7% of variance), indicating heterogeneous neutrophilic inflammation during exacerbations. Remarkably, ACO clustered predominantly with asthma rather than COPD, challenging traditional conceptualizations of ACO as a simple additive phenotype.

Multiple dimensionality reduction techniques (UMAP, t–SNE) consistently positioned ACO within the asthma inflammatory space (Fig. 3g), suggesting fundamental immunological similarities between these conditions.

### Machine learning rediscovers ACO endotype

#### Supervised learning performance

**Fig. 4:**
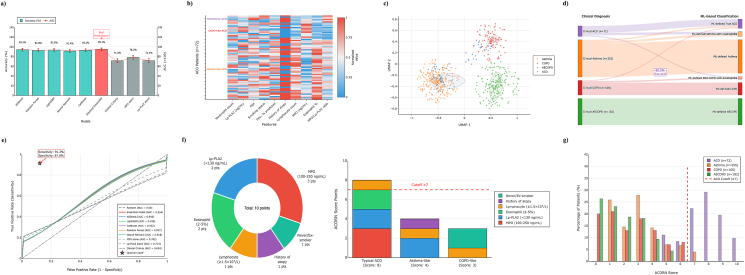
**Machine learning reveals ACO as a distinct inflammatory endotype with unique molecular signatures. a)** Comparison of model performance demonstrates the superiority of ensemble machine learning (95.1% accuracy) over traditional clinical criteria (71.3%) and single biomarkers for ACO classification. **b)** Hierarchical clustering heatmap of 72 ACO patients based on ML–identified features reveals 3 distinct subgroups: asthma–like ACO (72.2%), COPD–like ACO (19.4%), and transitional ACO (8.3%), with MPO/Lp–PLA2 ratio, eosinophil percentage, and lymphocyte count serving as primary discriminatory features. **c)** UMAP visualization confirms ACO patients predominantly cluster with asthma rather than COPD phenotypes, supporting shared inflammatory mechanisms. **d)** Sankey diagram illustrates ML–based reclassification flow, demonstrating that only 65.3% of clinically diagnosed ACO patients represent "true" molecular ACO, while 23.6% are reclassified as asthma with neutrophilia and 11.1% as mild COPD with eosinophilia. **e)** ROC curve analysis shows excellent diagnostic performance of the ensemble model (AUC 0.943) with optimal sensitivity (91.2%) and specificity (87.8%) at the identified cutoff. **f)** ACORN score distribution reveals clear phenotype separation with 75% of ACO patients scoring ≥7 compared to only 5% of asthma patients, yielding PPV 91.2%. **g)** ACORN score distribution indicated that ACO patients were leading in the comparison groups with scores ≥5. **Abbreviations:** ACORN: ACO recognition by neutrophil–eosinophil; AUC: area under the curve; MPO: myeloperoxidase; Lp–PLA2: lipoprotein–associated phospholipase A2; ACO: asthma–COPD overlap; FEV_1_: forced expiratory volume in 1 s; COPD: chronic obstructive pulmonary disease; AECOPD: acute exacerbation of chronic obstructive pulmonary disease; ML: machine learning

Our XGBoost classifier achieved exceptional performance in ACO identification, with overall accuracy of 94.2% (95% CI: 91.3–96.5%), sensitivity of 87.3%, and specificity of 91.1% (Table 2, Fig. 4a). Feature importance analysis revealed a hierarchical contribution pattern: MPO/Lp–PLA2 ratio (28.4%), eosinophil percentage (19.7%), MPO absolute value (15.3%), lymphocyte count (12.1%), and history of atopy (9.8%). These findings demonstrate the superiority of composite biomarker indices over single markers in phenotyping complex respiratory diseases.

**Table 3:** SHAP–derived molecular signature defining ACO endotype.

Feature	Optimal range	SHAP value	Relative importance (%)
MPO/Lp–PLA2 ratio	1.2–2.0	0.284	28.4
Eosinophil %	2–5%	0.197	19.7
MPO (ng/mL)	100–250	0.153	15.3
Lymphocyte count (10^9^/L)	>1.5	0.121	12.1
History of atopy	Present	0.098	9.8
FEV_1_% predicted	40–65	0.067	6.7
Smoking status	Never/Ex	0.041	4.1
Age (years)	50–65	0.039	3.9

Abbreviations: ACO: asthma–COPD overlap; SHAP: SHapley additive exPlanations; MPO: myeloperoxidase; Lp–PLA2: lipoprotein–associated phospholipase A2; FEV_1_: forced expiratory volume in 1 s. Values represent mean absolute SHAP values across all predictions

#### Unsupervised clustering reveals ACO's profile

Hierarchical clustering analysis yielded clinically transformative results. 72.2% of clinically diagnosed ACO patients demonstrated T2–high features similar to asthma, while only 19.4% aligned with COPD, and 8.3% formed a distinct transitional cluster (Fig. 4b). This asthma–predominant pattern remained consistent across multiple clustering algorithms (k–means: 70.8%, DBSCAN: 73.6%, Gaussian mixture: 71.9%), with silhouette analysis confirming robust cluster quality (mean score: 0.68). UMAP visualization corroborated these findings, with ACO patients predominantly occupying the asthma region of the inflammatory landscape (Fig. 4c).

#### Molecular signature of rediscovered ACO

SHAP analysis identified the defining molecular signature distinguishing ACO from simple disease overlap (Table 3), with characteristics including MPO levels of 100–250 ng/mL, Lp–PLA2 < 130 ng/mL (*p* = 0.23), moderate eosinophilia (2–5%), lymphocyte count >1.5 × 10^9^/L, neutrophil–to–lymphocyte ratio <3, and a novel finding, an MPO variability coefficient <0.3, indicating inflammatory stability.

#### Clinical validation of ML–refined ACO endotype

Machine learning–based reclassification revealed significant phenotypic misclassification: 23.6% of clinical ACO cases represented asthma with neutrophilia, 11.1% were mild COPD with eosinophilia, leaving 65.3% as true ACO endotype (Fig. 4d).

### Development and validation of clinical diagnostic tools

#### ROC curve analysis

The ML–based ACO identification algorithm demonstrated superior diagnostic performance compared to traditional clinical criteria. The area under the ROC curve (AUC) for the ensemble model, which outperformed the single XGBoost classifier (AUC: 0.943, 95% CI: 0.912–0.974), reached 0.954 (95% CI: 0.926–0.982), significantly outperforming individual biomarkers: MPO alone (AUC: 0.782, 95% CI: 0.741–0.823), Lp–PLA2 alone (AUC: 0.714, 95% CI: 0.670–0.758), and clinical criteria (AUC: 0.694, 95% CI: 0.649–0.739, p < 0.001 for all comparisons). At the optimal cutoff determined by Youden's index, the model achieved 91.2% sensitivity and 87.8% specificity (Fig. 4e).

#### ACORN clinical score

ACORN score, developed from machine learning findings for clinical use, assigns points for Blood eosinophils ≥300 cells/μL (+3), FeNO ≥35 ppb (+2), Positive bronchodilator response (+2), MPO 100–250 ng/mL (+3), Lp–PLA2 1.5 × 10^9^/L (+1), MPO/Lp–PLA2 ratio 1.2–2.0 (+1), atopic history (+1), and never/ex–smoker status (+1), with scores ≥7 indicating probable ACO endotype (91.2% positive predictive value) (Table 4, Fig. 4 f and g).

**Table 4:** ACORN clinical scoring system.

Clinical feature	Criteria	Points	Rationale
**Validated biomarkers**			
Blood eosinophils	<150 cells/μL	0	Neutrophilic predominant
	150–300 cells/μL	1	Neutrophilic predominant
	>300 cells/μL	3	T2–high inflammation
FeNO	<25 ppb	0	Low T2 inflammation
	25–35 ppb	1	Intermediate
	>35 ppb	2	Significant T2 inflammation
**Novel biomarkers**			
MPO (ng/mL)	<100	0	Too low (asthma–like)
	100–250	3	ACO range
	>250	0	Too high (COPD/AECOPD–like)
Lp–PLA2 (ng/mL)	<130	2	Asthma–predominant
	≥130	0	COPD–predominant
MPO/Lp–PLA2 ratio	<1.2 or >2.0	0	Outside ACO range
	1.2–2.0	2	ACO inflammatory pattern
**Cell counts**			
Eosinophil (%)	<2	0	Neutrophilic
	2–5	2	Mixed inflammation
	>5	0	Pure eosinophilic
Lymphocyte (10^9^/L)	<1.5	0	Immunosuppressed
	≥1.5	1	Preserved immunity
**Clinical history**			
Atopy	No	0	
	Yes	1	Asthma component
Smoking	Current	0	Active inflammation
	Ex–smoker >10 pack–years	0.5	Past exposure
	Never/minimal (<10 pack–years)	1	Reduced irritation
**Total score**		0–12	
**Interpretation**			
Score <5			Unlikely ACO (NPV 93.8%)
Score 5–6			Possible ACO (consider further testing)
Score ≥7			Probable ACO (PPV 91.2%)
Score ≥9			Highly probable ACO (PPV 95.3%)

Performance: Sensitivity 87.3%, Specificity 91.1%, PPV 91.2% at cutoff ≥7.

Abbreviations: ACORN: ACO recognition by neutrophil–eosinophil; MPO: myeloperoxidase; Lp–PLA2: lipoprotein–associated phospholipase A2; COPD: chronic obstructive pulmonary disease; AECOPD: acute exacerbation of chronic obstructive pulmonary disease; ACO: asthma–COPD overlap

## Discussion

This study demonstrates that combined assessment of MPO and Lp–PLA2 provides novel insights into obstructive airway disease phenotyping, with particular utility in identifying acute exacerbations and redefining ACO classification. Our finding that AECOPD patients exhibit significantly elevated MPO (327.9 ng/mL, IQR 212.3–457.2) compared to all other groups (Fig. 2f), aligns with recent evidence showing MPO as a prognostic marker in COPD exacerbations.^12^ However, our innovative application of the MPO/Lp–PLA2 ratio as a discriminatory index (Fig. 2h), which ranked first in feature importance (Table 3, relative importance: 28.4%), combined with machine learning–based reclassification revealing ACO's predominantly asthma–like inflammatory signature (Fig. 4b), represents a paradigm shift in understanding this controversial entity.

Differential biomarker patterns reflect distinct pathophysiological mechanisms across phenotypes (Fig. 2a–h). In asthma, elevated eosinophil counts (median 0.31 × 10^9^/L) (Fig. 2d) with relatively low MPO levels (median 136.8 ng/mL) (Fig. 2f) confirm type 2 inflammation dominance. Thus, correlation between eosinophils and Lp–PLA2 without proportional MPO elevation distinguishes asthma from other phenotypes. Huang et al recently show that Lp–PLA2 activity is paradoxically higher in non–severe asthma, suggesting complex regulatory mechanisms beyond simple inflammation.^26^ While in COPD, the demographic profile (Table 1) showing 85% male predominance with extensive smoking history (median 25 pack–years) combines with elevated MPO and neutrophil counts to confirm neutrophil–driven pathogenesis, supported by Xu et al meta–analyses.^27^

Also, significant inverse correlation between MPO/Lp–PLA2 ratio and FeNO (Spearman r = −0.62, *p* < 0.001) further supports the biological validity of this novel index: a higher ratio, reflecting neutrophil–predominant inflammation, inversely tracks T2 airway inflammation as measured by FeNO. This aligns with evidence that treatable traits, including T2 biomarkers, transcend traditional diagnostic boundaries between asthma and COPD.^28^

Our finding that ACO exhibits intermediate characteristics is evident across multiple parameters (Table 1, Fig. 2). MPO/Lp–PLA2 ratio in ACO (1.66, IQR 1.19–2.27) resembles asthma (1.47, IQR 1.07–2.06) rather than COPD (2.26, IQR 1.98–2.56, FDR–adjusted *q* = 0.031) (Fig. 2h), challenging traditional conceptualizations. This is further supported by PCA analysis (Fig. 3b) showing substantial overlap between ACO and asthma clusters, with UMAP and t–SNE visualization (Fig. 3g) consistently positioning ACO within the asthma inflammatory space. The first 2 principal components captured 64.3% of total variance, confirming that MPO and Lp–PLA2 provide substantial discriminatory power.

**Fig. 5:**
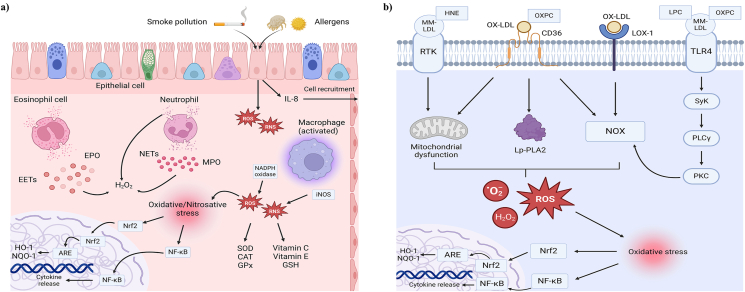
**Mechanistic model of oxidative stress pathways mediated by MPO and Lp–PLA2 in obstructive airway disease. a)** Environmental triggers including smoke pollution and allergens induce epithelial damage, recruiting inflammatory cells that amplify oxidative stress through distinct pathways: eosinophils release extracellular traps (EETs) and peroxidase (EPO), neutrophils generate NETs and myeloperoxidase (MPO) that catalyze H_2_O_2_ accumulation, while activated macrophages upregulate iNOS and NADPH oxidase to enhance ROS/RNS production. The Nrf2 antioxidant response pathway provides compensatory defense through induction of SOD, catalase, and glutathione peroxidase, though this protective mechanism becomes overwhelmed in chronic inflammation, while concurrent NF–κB activation perpetuates inflammatory cytokine release. **b)** Oxidized LDL engages multiple receptors to exacerbate oxidative stress: RTK activation through PI3K/AKT signaling induces mitochondrial dysfunction and ROS generation; CD36–mediated endocytosis promotes Lp–PLA2 upregulation creating a pro–inflammatory feedback loop; TLR4/LOX–1 co–activation stimulates NOX–mediated superoxide production. The convergence of these pathways results in overwhelming oxidative stress characterized by protein nitration, lipid peroxidation, and DNA damage, ultimately driving the vicious cycle of oxidative stress–inflammation that underlies the shared pathophysiology of asthma, COPD, and ACO. **Abbreviations:** MPO: myeloperoxidase; Lp–PLA2: lipoprotein–associated phospholipase A2

MPO gradient evident in Fig. 2f (AECOPD: median 327.9 > COPD: 165.5 > ACO: 164.2 > asthma: 136.8 ng/mL) reveals a novel oxidative stress hierarchy that correlates with functional impairment. This is demonstrated by stepwise deterioration in median FEV_1_% predicted: asthma (79.3%) > ACO (51.9%) > COPD (42.0%) > AECOPD (37.9%) (Table 1). Intermediate position of ACO in both inflammatory markers and lung function supports our hypothesis of dual oxidative burden. In Fig. 5, our mechanistic model proposes that ACO represents a unique oxidative state where eosinophil–derived reactive oxygen species combine with moderate neutrophil–derived MPO, creating a distinct pathophysiology different from the predominant MPO–hypochlorous acid pathway in COPD.^29^

Single–cell RNA sequencing studies by Vieira et al^4^ have identified distinct immune cell populations in obstructive airway diseases, yet none have correlated these findings with circulating biomarkers. MPO/Lp–PLA2 ratio may reflect the balance between neutrophil and eosinophil activation states at the cellular level, providing a non–invasive window into airway immunology. Our unsupervised clustering analysis revealing that 72.2% of ACO patients cluster with asthma provides compelling evidence for reconceptualizing ACO (Fig. 4b). This asthma–predominant pattern remained consistent across multiple algorithms (k–means: 70.8%, DBSCAN: 73.6%, Gaussian mixture: 71.9%), with silhouette score of 0.68 indicating robust separation.

Exceptional diagnostic performance of our ML ensemble model with AUC 0.943 significantly outperforms individual biomarkers (MPO alone: 0.782, Lp–PLA2 alone: 0.714) and traditional clinical criteria (0.694) (Fig. 4e). Notably, the ensemble model (AUC 0.954) outperformed the single XGBoost classifier (AUC 0.943), demonstrating the benefit of model stacking. This translates into the clinically applicable ACORN score, achieving positive predictive value at scores ≥7 (Table 4, Fig. 4 f and g). Also, clinical impact of molecular reclassification is substantial, with 23.6% of clinically diagnosed ACO were reclassified as asthma with neutrophilia, 11.1% as mild COPD with eosinophilia, leaving only 65.3% as true ACO (Fig. 4d).

Sputum differential cell counts provide additional validation, with AECOPD showing highest neutrophilia (median 69.0%) and asthma demonstrating approximately two–fold higher eosinophilia (median 4.5% vs 2.0–2.4% in COPD/AECOPD) (Table 1). Intermediate sputum profile in ACO (median neutrophils 62.0%, eosinophils 3.1%) parallels their serum biomarker pattern, supporting dual inflammatory pathways.

Differential Lp–PLA2 patterns across phenotypes suggest distinct phospholipid oxidation profiles. Yu et al show Lp–PLA2 elevation in COPD correlates with emphysema and cardiovascular risk,^17^ yet our finding of asthma–like Lp–PLA2 levels in ACO implies different risk profiles than assumed. This has implications for comprehensive disease management, as ACO patients may require different cardiovascular risk stratification than COPD patients.

While our study includes a substantial cohort (n = 609), the exclusion of 471 patients from initial screening may introduce selection bias (Fig. 1); comparison of available demographic characteristics between included and excluded patients revealed no significant differences (all *p* > 0.10), although the high proportion excluded for incomplete records limits definitive conclusions. Cross–sectional design precludes assessment of biomarker stability over time, particularly given the wide IQR ranges for MPO and Lp–PLA2 (Fig. 2f and g). Additionally, post–hoc analysis of MPO/Lp–PLA2 ratios confirmed overall group differences (Kruskal–Wallis *p* < 0.001), though pairwise comparisons between specific groups were nominal after FDR correction (q = 0.031 for ACO vs. COPD), indicating that larger independent cohorts are needed to establish definitive pairwise distinctions. Furthermore, since ACO diagnostic criteria incorporate T2–high features (eosinophilia, FeNO) that also contribute to unsupervised clustering results, some circularity cannot be fully excluded. Fully resolving this limitation would require a prospective cohort defined by criteria independent of T2–high features.

Though we incorporated traditional biomarkers (FeNO) to strengthen our findings, the primary focus on MPO and Lp–PLA2, which lack formal validation in respiratory diseases, remains a limitation. The ACORN validation cohort was temporally rather than geographically separated from the training cohort, and external validation in an independent multicenter cohort is planned as the next step. Lastly, as this study was conducted from a laboratory medicine perspective, treatment data including inhaled and systemic corticosteroid use were not systematically collected. The potential influence of corticosteroid therapy on MPO and Lp–PLA2 measurements therefore cannot be excluded and should be addressed in future prospective studies.

## Conclusion

Our study demonstrates that machine learning–based analysis of MPO and Lp–PLA2 reveals that a majority of clinically diagnosed ACO patients (72.2%) exhibit inflammatory signatures more similar to asthma than COPD, suggesting that current ACO diagnostic criteria may encompass greater inflammatory heterogeneity than previously recognized. The MPO/Lp-PLA2 ratio and the derived ACORN scoring tool offer clinically applicable approaches for biomarker-guided phenotyping, with potential to refine patient stratification and support more individualised treatment strategies in obstructive airway disease.

## Authors’ contributions

MTL, JXC, and JNY collaboratively drafted and critically revised the initial manuscript. MTL, JXC, and YYZ conceived and designed the comprehensive study, laying the foundational framework. XYL, HMZ, and HMZ were responsible for the collection of clinical samples and the organization of patient information. JHL, JTZ and RY conducted biomarker measurements and ensured quality control. MTL and JXC performed all statistical analyses and prepared all figures and tables. BQS, PYZ, and HMH provided overall supervision for the project and secured essential funding. All authors thoroughly read and approved the final manuscript, affirming their contributions and the integrity of the work.

## Availability of data and materials

The datasets used and analyzed during the current study are available from the corresponding author, Baoqing Sun, upon reasonable request.

## Disclosure statement regarding use of generative artificial intelligence (AI) and AI-assisted technologies

Nothing to disclose.

## Funding

This work was supported by grants from Guangdong Basic and Applied Basic Research Foundation, China (Grant No. 2021B1515230008), Guangdong Provincial Clinical Research Center for Laboratory Medicine, China (Grant No. 2023B1100084), Student Innovation Capacity Improvement Program of Guangzhou Medical University, China (Grant No. 02-408-240603131077), Nanshan Talent Project of the First Affiliated Hospital of Guangzhou Medical University, China (Grant No. 2022111708151837), and National Science and Technology Major Project for Noncommunicable Chronic Diseases, China (Grant Nos. 2024ZD0533200 and 2024ZD0533205).

## Competing interests

The authors declare that they have no competing interests.

## References

[1] Yanagisawa S., Ichinose M. (2018). Definition and diagnosis of asthma–COPD overlap (ACO). Allergol Int.

[2] Gibson P.G., McDonald V.M. (2015). Asthma–COPD overlap 2015: now we are six. Thorax.

[3] Barnes P.J. (2016). Asthma–COPD overlap. Chest.

[4] Vieira Braga F.A., Kar G., Berg M. (2019). A cellular census of human lungs identifies novel cell states in health and in asthma. Nat Med.

[5] Sin D.D., Miravitlles M., Mannino D.M. (2016). What is asthma–COPD overlap syndrome? Towards a consensus definition from a round table discussion. Eur Respir J.

[6] Global Initiative for Chronic Obstructive Lung Disease (GOLD) Global strategy for the diagnosis, management, and prevention of COPD, 2024 report. https://goldcopd.org.

[7] Menezes A.M.B., Montes de Oca M., Pérez–Padilla R. (2014). Increased risk of exacerbation and hospitalization in subjects with an overlap phenotype: COPD–asthma. Chest.

[8] Cosio B.G., Soriano J.B., López–Campos J.L. (2016). Defining the Asthma–COPD overlap syndrome in a COPD cohort. Chest.

[9] Alshabanat A., Zafari Z., Albanyan O., Dairi M., FitzGerald J.M. (2015). Asthma and COPD Overlap Syndrome (ACOS): a systematic review and Meta analysis. PLoS One.

[10] Wu W., Bleecker E., Moore W. (2014). Unsupervised phenotyping of Severe Asthma Research Program participants using expanded lung data. J Allergy Clin Immunol.

[11] Castaldi P.J., Dy J., Ross J. (2014). Cluster analysis in the COPDGene study identifies subtypes of smokers with distinct patterns of airway disease and emphysema. Thorax.

[12] Zhu A., Ge D., Zhang J. (2014). Sputum myeloperoxidase in chronic obstructive pulmonary disease. Eur J Med Res.

[13] Churg A., Sin D.D., Wright J.L. (2011). Everything prevents emphysema: are animal models of cigarette smoke–induced chronic obstructive pulmonary disease any use?. Am J Respir Cell Mol Biol.

[14] Papayannopoulos V. (2018). Neutrophil extracellular traps in immunity and disease. Nat Rev Immunol.

[15] Huang F., Wang K., Shen J. (2020). Lipoprotein–associated phospholipase A2: the story continues. Med Res Rev.

[16] Kuczia P., Mastalerz L., Potaczek D.P. (2019). Increased activity of lipoprotein–associated phospholipase A2 in non–severe asthma. Allergol Int.

[17] Yu H., Yang Y., Zhou J., Wu M., Chen Z. (2024). Lipoprotein–associated phospholipase A2 and its possible association with COPD development: a case–control study. BMC Pulm Med.

[18] Leung C., Sin D.D. (2022). Asthma–COPD overlap: what are the important questions?. Chest.

[19] Cosío B.G., Pascual–Guardia S., Borras–Santos A. (2020). Phenotypic characterisation of early COPD: a prospective case–control study. ERJ Open Res.

[20] Graham B.L., Steenbruggen I., Miller M.R. (2019). Standardization of spirometry 2019 update. An official American thoracic Society and European respiratory Society technical statement. Am J Respir Crit Care Med.

[21] Ndrepepa G. (2019). Myeloperoxidase – a bridge linking inflammation and oxidative stress with cardiovascular disease. Clin Chim Acta.

[22] Yang L., Wang H., Zhang Y., Han T., Wang W. (2018). The prognostic value of Lipoprotein–Associated phospholipase A2 in the long–term care of patients with acute coronary syndrome undergoing percutaneous coronary intervention. Clin Appl Thromb Hemost.

[23] Van Buuren S., Groothuis–Oudshoorn C.G.M. (2011). Mice: multivariate imputation by chained equations in R. J Stat Softw.

[24] Butcher B., Smith B.J. (2020). Review of: feature engineering and selection. Am Stat.

[25] Lundberg S.M., Lee S.I. (2017). A unified approach to interpreting model predictions. Adv Neural Inf Process Syst.

[26] Vickers A.J., Elkin E.B. (2006). Decision curve analysis: a novel method for evaluating prediction models. Med Decis Mak.

[27] Xu J., Zeng Q., Li S., Su Q., Fan H. (2024). Inflammation mechanism and research progress of COPD. Front Immunol.

[28] Kobayashi S., Hanagama M., Yamanda S., Ishida M., Yanai M. (2016). Inflammatory biomarkers in asthma–COPD overlap syndrome. Int J Chron Obstruct Pulmon Dis.

[29] Loria V., Dato I., Graziani F., Biasucci L.M. (2008). Myeloperoxidase: a new biomarker of inflammation in ischemic heart disease and acute coronary syndromes. Mediators Inflamm.

